# Effect of in utero and lactational exposure to antiretroviral therapy on the gut microbial composition and metabolic function in aged rat offspring

**DOI:** 10.3389/ebm.2025.10468

**Published:** 2025-05-21

**Authors:** Chandra Mohan Reddy Muthumula, Yaswanthi Yanamadala, Kuppan Gokulan, Kumari Karn, Helen Cunny, Vicki Sutherland, Janine H. Santos, Sangeeta Khare

**Affiliations:** ^1^ Division of Microbiology, National Center for Toxicological Research, US Food and Drug Administration, Jefferson, AR, United States; ^2^ Division of Translational Toxicology, National Institute of Environmental Health Sciences, Research Triangle Park, NC, United States

**Keywords:** antiretroviral therapy, intestine, fecal, microbiome, short-chain fatty acids, IgA, perinatal exposure

## Abstract

Despite the highly effective impact of antiretroviral therapy (ART) in reducing mother-to-child transmission of human immunodeficiency virus (HIV), there are concerns of long-term impacts of ART on the health of the offspring. The implications of perinatal exposure to antiviral drugs on the gut bacterial population and metabolic function in the offspring is unclear but may influence health outcomes given the various reported effects of the microbiome in human health. This study aims to gain insight into the potential effect of *in utero* and lactational exposure to ART on gut microbiota populations and short‐chain fatty acids (SCFAs) production in aged rat offspring. Pregnant rats were administered a combination of antiretroviral drugs (abacavir/dolutegravir/lamivudine) at two different dose levels during gestation and throughout lactation, and the fecal bacterial abundance and SCFA levels of the offspring were analyzed when they reached 12 months of age. Our results showed dose-dependent and sex-based differences in fecal microbial abundance at various taxonomic levels. Specifically, we found a decline in *Firmicutes* in males, and an increase in *Actinobacteria* among males and females. Furthermore, a sex-specific distribution reorganization of *Lactobacillus*, *Bifidobacterium*, and *Akkermansia* was identified. No significant difference in the concentration of prominent SCFAs and IgA levels were identified. These findings provide preliminary information indicating the need to evaluate perinatal effects of ART more comprehensively on the gut bacterial and metabolic function in future studies, and their potential role in offspring health outcomes.

## Impact statement

This work addresses a critical health challenge on understanding how preventive HIV medications given during pregnancy could affect complex community of gut bacteria in next (f1) generation using a rat model. The work advances our understanding by analyzing both gut bacterial communities and their products in aged rat offsprings including sex-specific responses. This study showed that early exposure to these medicines lead to changes in gut bacterial composition. In addition, this effect also differed between male and female rats. However, their metabolic products (short chain fatty acids) and immune factors (IgA) remained stable. These findings impact the field by highlighting the importance of inclusion of male and female as a biological factor. This study provides a foundation for understanding how early exposure to HIV medications might influence long-term development and suggest new directions for monitoring offspring health.

## Introduction

The gut microbiome consists of a wide network of microorganisms (including various types of bacteria, fungi, archaea, and viruses) that live in the gastrointestinal tract [1]. This gut microbiome plays a major role in regulating the health of an individual. This complex system is involved in various essential physiological processes including nutrient metabolism, development of the immune system, and protection against pathogenic microorganisms [2]. The composition and function of the gut microbiome is influenced by a variety of factors, including diet, use of antibiotics, and the host genetics [3]. When an unhealthy imbalance occurs in the gut microbial composition, it can lead to various metabolic diseases and health problems such as obesity, type 2 diabetes, and inflammatory bowel disease [4].

Various studies have shown the role of maternal microbiome as a key determinant of the offspring’s gut microbiome composition and function [5–9]. During pregnancy and childbirth, microbial populations are passed from mother-to-child through vertical transmission with the mode of delivery (vaginal birth vs. cesarean section) and feeding practices (breastfeeding vs. formula feeding) influencing the initial colonization of the infant gut [5–9]. This early-life colonization of the microbial communities influences the long-term development of the offspring’s health outcomes, such as the maturation of the immune system, development of metabolic pathways, establishment of the gut-brain axis, etc. [10–12]. Studies have shown that disruptions to this early microbial colonization have been linked to an increased risk of allergies, asthma, and metabolic disorders later in life [13].

Abacavir, dolutegravir, and lamivudine (a combination of three antiretroviral drugs) have been used in the management of Human Immunodeficiency Virus (HIV) infection in both adult and pediatric patients. This tri-combination antiretroviral therapy (ART) consists of drugs from 2 different classes: nucleoside reverse transcriptase inhibitors (abacavir and lamivudine) and HIV integrase inhibitors (dolutegravir) [14–16]. In HIV-infected pregnant women, ART is essential for preventing transplacental (mother-to-fetus) transmission of HIV infection [17–20]. The long-term side effects of ART may be under rated if the clinical trials utilize very specific inclusion/exclusion criteria, and the follow-up duration is relatively short [21]. Numerous studies suggest that ART may affect the composition and diversity of the gut microbiome [22, 23]. Alteration in the maternal microbiome could potentially influence the vertical transmission of microbial communities to the offspring [24]. In addition, HIV infection itself has been shown to disrupt the normal balance of microorganisms in the gut, characterized by a decrease in beneficial bacteria (such as *Bacteroides*) and an increase in potentially pathogenic ones (such as Prevotella) [25, 26]. While ART is essential for managing HIV infection, its effect on gut microbial diversity is not clear. Some studies suggest it helps restore diversity while other studies indicate that ART further disrupts the gut microbial diversity. Since many factors like immune health, diet etc., influence the microbiome, it is unclear whether ART may worsen or alleviate the alterations in the gut microbial composition on both the maternal and infant microbiome [25, 26].

In addition to the various physiological functions, gut bacterial populations are also involved in the production of various short‐chain fatty acids (SCFAs). These SCFAs (such as acetate, propionate, and butyrate, etc.) are key microbial metabolites produced from the fermentation of dietary fibers [27]. SCFAs have been shown to exert various beneficial effects on host health, including the regulation of immune function, energy metabolism, and gut barrier integrity [28–30]. Various studies have shown that alterations in SCFA production lead to the development of various metabolic diseases, including cardiovascular disease, obesity, and type 2 diabetes [31–36]. ART-induced disruption of the microbial ecosystem may alter SCFA production, potentially influencing the development and function of the infant gut microbiome and leading to long-lasting health consequences for the offspring. However, the specific impact of gestational ART exposure on SCFA production in the offspring remains largely unexplored.

Fecal bacterial population profiling has emerged as a powerful non-invasive tool for assessing the composition and function of the distal gut microbial community [37, 38]. Various studies reported that high-throughput sequencing technologies such as 16S rRNA gene sequencing and shotgun metagenomics, are useful to obtain a comprehensive snapshot of the microbiome composition and its functional potential [39, 40]. Furthermore, integrating metabolomic analysis such as SCFA quantification, with taxonomic profiling has provided valuable insights into the complex interplay between the gut microbiome and host physiology [41, 42]. However, limited studies have applied these multi-omics approaches to investigate the long-term impact of perinatal ART exposure on the offspring gut microbiome and metabolome.

In addition to the complex interplay between the gut microbiome and ART, it is also important to consider the role of the immune system in shaping the microbial composition. Immunoglobulin A (IgA), plays an important role in maintaining the delicate balance between the immune system and the gut microbiota of the host, ensuring a mutually beneficial relationship [43]. Investigating the interplay between gut microbiome, IgA, and SCFAs in the context of perinatal ART exposure could provide valuable insights into the balance between the immune system and microbial metabolites that could influence the offspring’s health.

The current study tested whether indirect exposure through the Dams to Abacavir Sulfate (ABC)/Dolutegravir Sodium (DTG)/Lamivudine (3TC), hereon called TC-ART, led to changes in the gut microbiome when the offspring reached 1 year of age. In addition, changes in the abundance and activity of SCFA-producing bacteria resulting in altered SCFA profiles in the offspring, were examined.

### Rationale for selection of antiretroviral treatment regimen

The drug regimen consisting of abacavir, dolutegravir, and lamivudine, was selected for this study based on being the current recommended TC-ART to be provided during pregnancy for patients that are naïve to ART or already on this combination^1^. For adults and children weighing 25 kg or more, this TC-ART is administered at a dosage of 600-50-300 mg in tablet form once daily [44, 45]. The selection of this once-daily combination therapy in the rat model is expected to mimic the dosing schedule used in human patients.

## Materials and methods

### Animal housing, care, treatment, euthanasia, and sample collection

**FIGURE 1:**
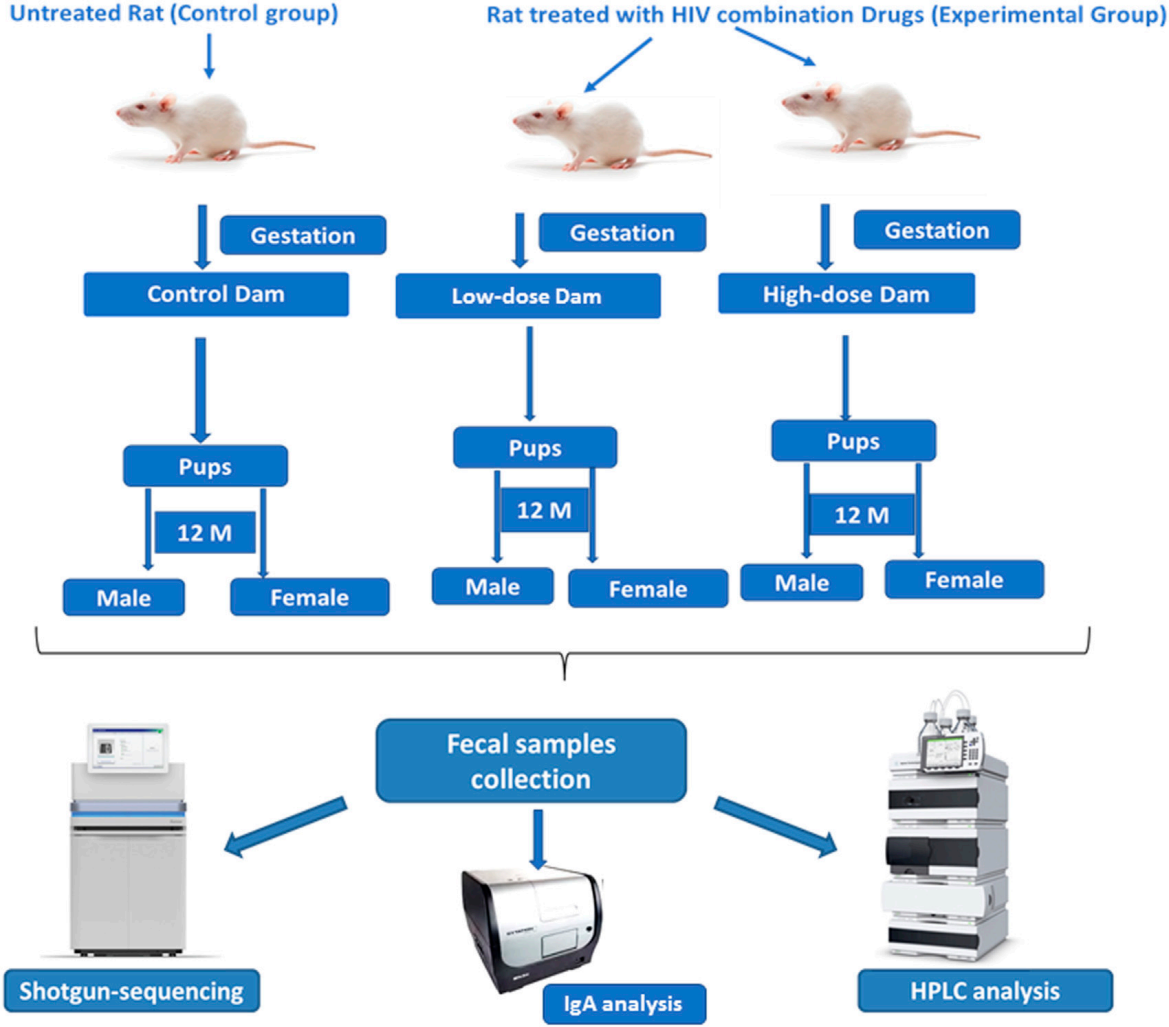
Experimental design for evaluating the effects of perinatal exposure of the HIV Tri-combination drug in a rat model (F1 generation). Schematic representation of the experimental design for 12-month-old F1 generation study shows that the pregnant female rats (dams) were divided into control (untreated) and experimental groups (receiving low- and high-dose HIV tri-combination drugs) during gestation till post-partum day 21. Male and female offspring were monitored for 12 months (F1 generation). Fecal samples were collected from all the offspring and analyzed using shotgun sequencing, HPLC, and IgA profiling to access microbiome composition, metabolite profiles, and mucosal immunity.

Time mated Sprague Dawley rats (Hsd:SD) were obtained from Envigo (Indianapolis, IN). Pregnant rats and their male and female offspring were housed in the animal facility at Amplify Bio, West Jefferson, OH, an independent, scientific contract research organization. The facility’s Institutional Animal Care and Use Committee (IACUC) reviewed the protocol and approved it. The IACUC number for this protocol is T06055. All rats were housed in polycarbonate cages with irradiated hardwood bedding chips (Sani Chips^®^; Envigo, Madison, WI). Natural crinkled kraft paper was provided during gestation and lactation for enrichment (Crink-l’nest™, The Andersons, Maumee, Ohio). Offspring remained with their respective dams until postnatal day (PND) 21. After the lactation period, first generation offspring were provided polycarbonate rectangular shelters (Rat Retreats™, Bio-Serve, Flemington, NJ) as enrichment and were group housed by sex, up to 5 per cage. Animals were fed irradiated NIH-07 pellets or wafers (Zeigler Bros., Gardners, PA) during gestation and lactation. After weaning, rats were fed NTP-2000 (Zeigler Bros., Gardners, PA). All animals were provided municipal water *ad libitum* from an automatic watering system. The water and feed were analyzed for known contaminants that could interfere with or affect the outcome of the study, and none were found. The animals used in this study of microbiome were part of a larger toxicology study that will be reported separately. The experimental design for the microbiome investigation is outlined in the Figure 1.

Pregnant Sprague Dawley rats (n = 5/group) were exposed via gavage to two different doses of the TC-ART (abacavir/dolutegravir/lamivudine) during gestation and lactation (GD6 - PND21). The doses of TC-ART used in this study were a low-dose of 150/12.5/75 mg/kg body weight and a high-dose of 300/25/150 mg/kg body weight. All animals, including the control group, were administered vehicle solution (0.2% methylcellulose/0.1%, Tween 80) at the same volume (5 mL/kg) and frequency as treatment groups. The offspring were indirectly exposed to the ART via the dam during their perinatal period only. The offspring (one male and female from each dosed or control dam) were aged to 12 months with no direct dosing. At the age of 12 months, these aged rats were sacrificed. Fecal samples were collected aseptically from the colon of animals and immediately transferred to liquid nitrogen and thereafter kept frozen at −80°C for the assessment of gut microbiota, SCFAs, and fecal IgA (bound and unbound) (Figure 1).

### Fecal DNA extraction and long read sequencing

The DNA and RNA from the rat fecal samples were isolated using Zymo ZR-duet DNA/RNA Miniprep (Zymo Research, Tustin, CA, United States) as per manufacturer instructions. The quality and quantity of DNA was checked using a Cytation3 Cell Imaging Multimode Reader (BioTek, Winooski, VT, United States) and QubitTM Fluorometer (Thermo Fisher Scientific, Waltham, MA, United States).

### Microbiome sequencing and analysis

To investigate the fecal microbiota composition of control, low-dose, and high-dose treated groups in both male and female rats, Illumina NovoSeq sequencing technology was utilized. Operational Taxonomic Units (OTUs) were generated for each sample using a 97% sequence similarity threshold, and the number of sequences in each OTU was determined. The OTU representative sequences were compared against a microbial reference database to obtain classification information for each species corresponding to each OTU. Microbiome diversity and community structure were assessed via shotgun sequencing, using libraries prepared with a procedure adapted from the Nextera XT Kit (Illumina). Sequencing was performed on an Illumina NovaSeq 6000 platform, with paired-end 2 × 150 sequencing and a target depth of 20 million reads. DNA sequences were filtered for low quality (Q-Score <30) and length (<50 bp), and adapter sequences were trimmed using Cutadapt. Host sequences were removed using Bowtie2. Bacterial 16S rRNA gene sequences were extracted from the shotgun data and used for the microbiome analysis. Using the web-based platform MicrobiomeAnalyst [46, 47]. The Greengenes database was used for taxonomic classification. Data filtering included the removal of low-count features with a minimum count of 4 and a prevalence of 20% in samples, as well as low-variance features with a 10% cutoff. Data normalization was performed using Total Sum Scaling (TSS). Rarefaction curves were used to evaluate sequencing depth and Good’s index was used to assess sequencing completeness. Alpha diversity was assessed using 2 metrics: Chao1 and Shannon index. Analysis of variance (ANOVA) was used to determine statistically significant differences in microbial community diversity in response to TC-ART treatment. Beta diversity was analyzed using Principal Coordinate Analysis (PCoA) based on Bray-Curtis dissimilarity. Similarity analysis was conducted using Euclidean distance and the Ward hierarchical clustering algorithm, with results presented in a heatmap.

### Fecal sample collection and processing for SCFA using HPLC

For SCFA profiling, 100 mg of frozen feces was weighed out into microcentrifuge tubes. One milliliter of chilled HPLC-grade water was added to the respective tubes. The samples were vortexed for 1 min and sonicated for 10 min until homogenized. The homogenized samples were then centrifuged at 18,000 × g for 10 min at 4°C. The supernatant was collected and filtered through a 0.22 μm syringe filter into amber HPLC vials.

### Quantification of SCFA

SCFAs were quantified using high-performance liquid chromatography (HPLC) analysis. An Agilent Technology 1260 Infinity system coupled with an Agilent Technology Infinity Lab LC/MSD mass spectrophotometer and an auto sampler system was used for the analysis. Chromatographic separation and identification of SCFAs were performed using an Aminex HPX-87H column (300 mm × 7.8 mm, hydrogen form, 9 μm particle size, 8% cross-linkage; Bio-Rad) maintained at 65°C. A UV detector set at 210 nm using a spectral diode array system was employed for detection. The mobile phase consisted of freshly prepared 2.5 mM H_2_SO_4_ with a flow rate of 0.6 mL/min. The sample injection volume was set to 10 μL.

Calibration standards were prepared by diluting the respective reference standards for the following SCFAs: succinic acid, lactic acid, formic acid, acetic acid, propionic acid, isobutyric acid, butyric acid, isovaleric acid, valeric acid, hexanoic acid, and heptanoic acid in 2.5 mM H_2_SO_4_. Standards, samples, and spiked samples were analyzed by HPLC, and SCFAs were identified and quantified by retention time and peak area relative to the standards. The percentage recovery of the SCFAs from extraction ranged between 80.83 and 92.15%.

### Quantification of bound and unbound IgA in rat feces

To quantify the level of bound and unbound IgA in rat feces, we followed the procedure described by Lahiani at al [48]. Rat fecal samples were weighed and diluted to prepare a concentration of 50 mg/mL PBS buffer containing 1mM phenylmethylsulphonyl fluoride (PMSF) and 1mM protease inhibitor cocktail solution. The samples were then vortexed rigorously and centrifuged at 4°C for 15 min at a speed of 900g. The resulting supernatant was collected and filtered through 0.22 um PTFE syringe filters to measure the unbound IgA level.

For the collection of bacterial-bound IgA, the same filter was washed with 0.05% tween 20, and the flow through was collected. A Rat IgA ELISA Kit (Bethyl Laboratories, Montgomery, TX, United States) containing pre-coated 96 well strip plate was used to assess the levels of IgA according to the manufacturers protocol. The absorbance was measured on a Cytation 3 plate reader (BioTek) at 450 nm. The standard curve was fitted into a 4-parameter curve fitting equation to calculate the analyte concentration in the original sample.

### Statistical analysis of SCFAs and IgA levels

For comparing SCFAs and IgA levels between treatment groups, an unpaired two-sample t-test was performed. The significance was set at 5% (p ≤ 0.05). The t-test assessed differences between the means of the data sets by calculating the variance from all animals in each group (n = 5). P-values below 0.05 were considered statistically significant.

## Results

### Diversity analysis of fecal microbial communities across treatment groups

#### Alpha diversity analysis

Alpha diversity measures the richness and diversity of species within a single sample using various indices, such as Chao1 and Shannon. The Chao1 index assesses species richness (i.e., the number of species), while the Shannon index evaluates species diversity, considering both richness and community evenness. In this study, the completeness of sequencing was tested using Good’s coverage, which reached 100%, indicating that the majority of the bacterial species present in the samples had been detected.

**FIGURE 2:**
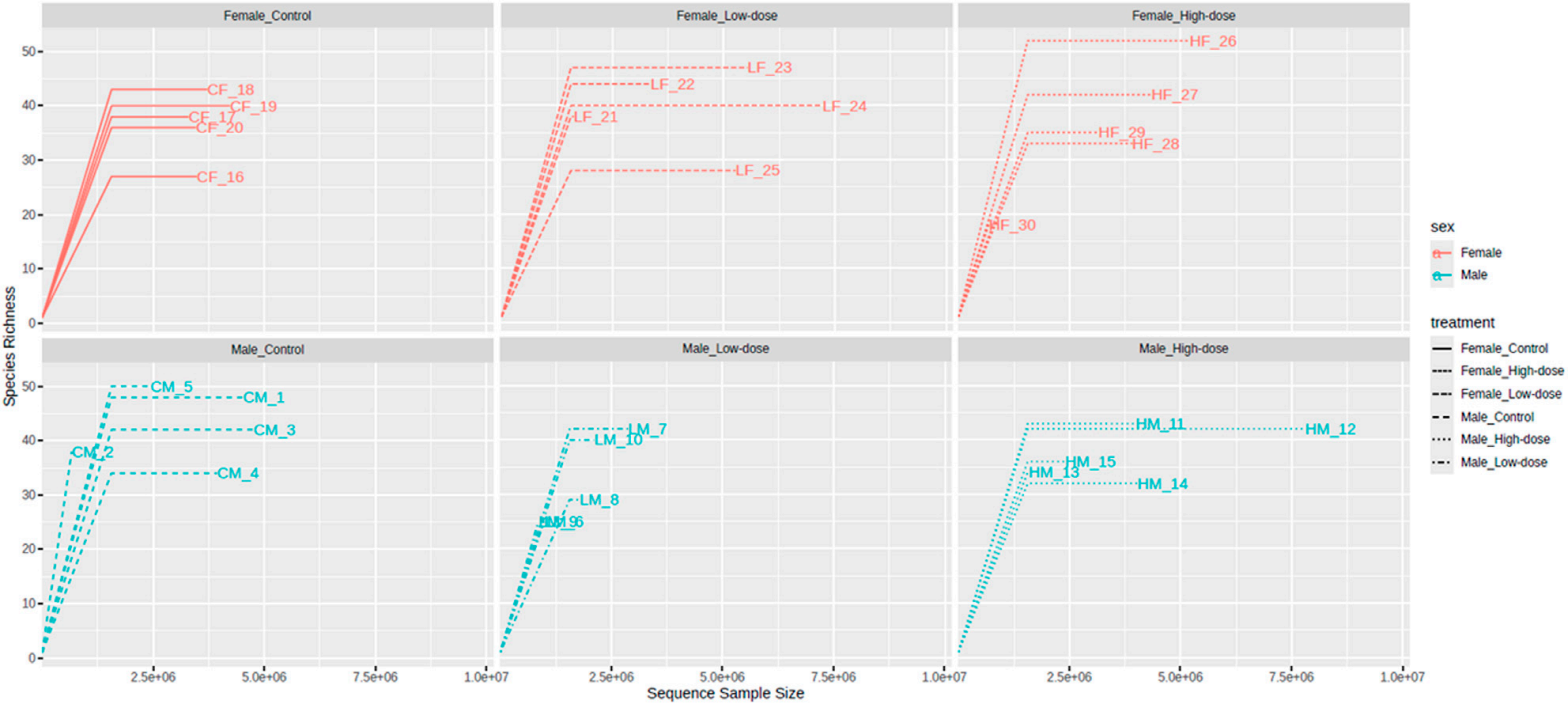
Rarefaction curves of the observed Operational Taxonomic Units (OTUs) in the fecal microbiome across different treatment groups (control, low-dose, and high-dose) in both male and female rats. The rarefaction curves plot the number of observed OTUs as a function of the number of sequences sampled, with a plateauing curve indicating that the majority of the bacterial species present have been captured.

The rarefaction curves of the observed OTUs (Figure 2) revealed that the number of OTUs increased with sequencing depth for all treatment groups. In females, the control (26–39 OTUs), low-dose (23–41 OTUs), and high-dose (17–47 OTUs) groups all showed substantial overlap, preventing clear discrimination of diversity changes due to treatment. Similar results were obtained for males: the control (32–51 OTUs), low-dose (25–38 OTUs), and high-dose (31–42 OTUs). The stabilization of the final curve indicates that the amount of sequencing data obtained was sufficient and representative.

**FIGURE 3:**
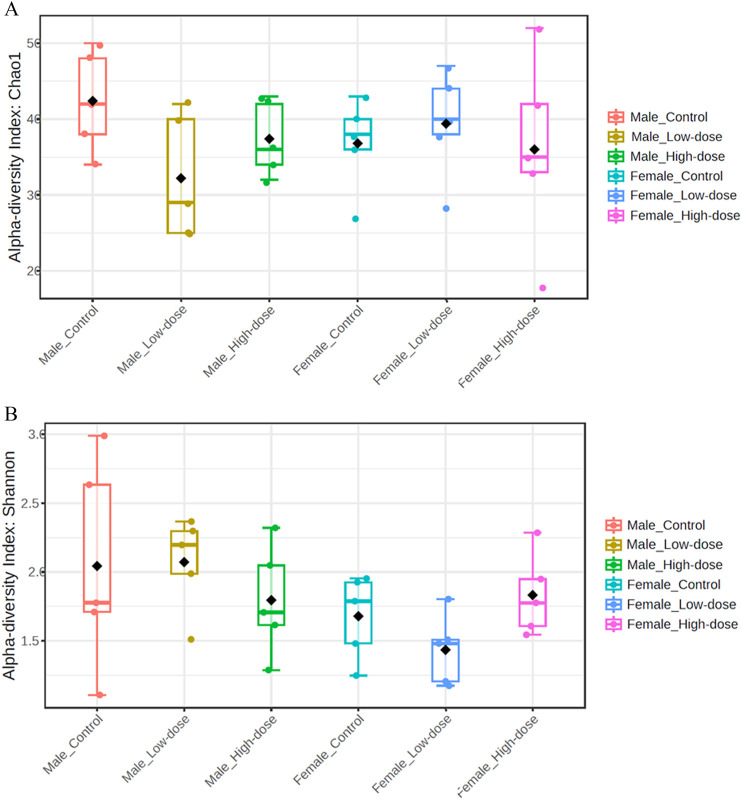
Fecal microbial population diversity across different treatment groups (control, low-dose, and high-dose) in both male and female rats. **(A)** Alpha diversity indices (Chao1) assess species richness, with higher values indicating a greater number of unique species within a sample. **(B)** Alpha diversity indices (Shannon) of the fecal microbiome across different treatment groups (control, low-dose, and high-dose) in both male and female rats assess species diversity, taking into account both richness and evenness, with higher values indicating greater diversity within a sample.

The Chao1 index values (Figure 3A) ranged from 18 to 52 across all samples, with the highest value observed in the female high-dose group (HF_26) and the lowest in the female high-dose group (HF_30). No significant differences were observed when comparing the Chao1 index across groups, suggesting that the treatment did not significantly impact species richness in either male or female rats.

The Shannon index values (Figure 3B) ranged from 1.11 to 2.99, with the highest value found in the male control group (CM_5) and the lowest in the male control group (CM_4). Similar to the Chao1 index, no significant differences were observed in the Shannon index across treatment groups.

Collectively, these data demonstrate that TC-ART treatment did not alter species richness or diversity in aged male and female rats perinatally exposed to these drugs.

#### Comparison of fecal microflora across treatment groups

The identified bacteria were categorized into 7 phyla, 11 classes, 14 orders, 26 families, 39 genera, and 68 species across the animals. The composition of each sample community was calculated at every taxonomic level (phylum, class, order, family, genus, and species). Table 1 represents the taxonomic level classification of individual samples.

**TABLE 1:** Operational Taxonomic Units (OTUs) species of samples on various Taxonomic levels.

Sample	Kingdom	Phylum	Class	Order	Family	Genus	Species
CM-1	1	6	9	11	20	27	48
CM-2	1	6	9	9	15	23	38
CM-3	1	6	8	10	19	26	43
CM-4	1	5	7	8	16	21	34
CM-5	1	6	10	11	21	29	53
LM-1	1	7	9	10	16	21	26
LM-2	1	6	10	11	19	27	42
LM-3	1	6	9	10	17	23	29
LM-4	1	5	7	8	14	20	25
LM-5	1	5	8	9	18	25	40
HM-1	1	6	10	11	20	27	43
HM-2	1	6	10	11	20	26	43
HM-3	1	6	9	10	18	24	34
HM-4	1	5	7	8	15	19	32
HM-5	1	5	8	9	17	23	37
CF-1	1	4	6	7	15	19	27
CF-2	1	6	8	9	18	24	38
CF-3	1	6	10	11	20	26	43
CF-4	1	5	7	8	19	24	41
CF-5	1	5	8	9	16	22	36
LF-1	1	5	9	10	18	23	38
LF-2	1	5	9	10	20	26	44
LF-3	1	5	9	10	21	29	47
LF-4	1	5	9	10	21	25	40
LF-5	1	5	7	8	15	18	28
HF-1	1	6	10	12	23	31	52
HF-2	1	6	10	11	21	25	42
HF-3	1	5	7	8	16	20	33
HF-4	1	5	8	9	17	23	35
HF-5	1	6	8	9	13	16	18

Table represents taxonomic diversity across control and experimental groups (CM, Control Male; LM, low-dose Male; HM, high-dose Male; CF, Control Female; LF, low-dose Female; HF, high-dose Female) followed by replicate numbers (1–5). Numbers indicate distinct taxonomic units detected at each classification levels from Kingdom to species.

#### Phylum level analysis

**FIGURE 4:**
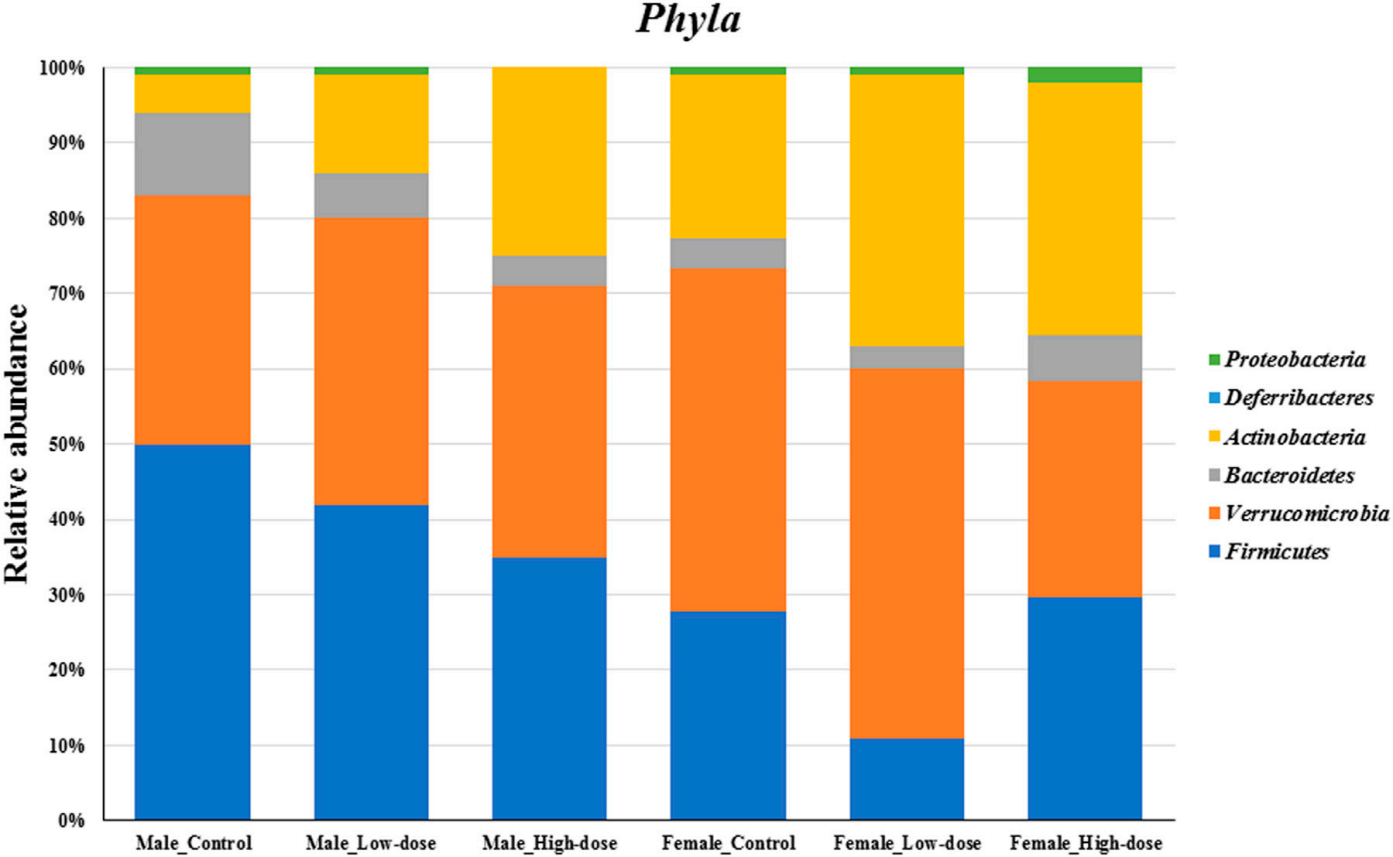
Changes in the fecal microbial composition at the *Phyla* level (top six phylum) across different treatment groups (control, low-dose, and high-dose) in both male and female rats (n = 5 in each group).

At the phylum level (Figure 4), differences in relative abundance were observed between males and females in the control group (compare first and fourth bar on Figure 4), and further changes were observed upon treatment. Specifically, the relative abundance of both *Firmicutes* and *Bacteroidetes* exhibited a dose-dependent decrease in males, with more pronounced effect in the high-dose group. Conversely, a dose-dependent increase was observed for *Actinobacteria*. In females, changes in relative abundance did not follow dose-dependency, with *Firmicutes* decreasing in the low-dose but increasing in the high-dose group compared to the control. Similarly, *Actinobacteria* relative levels were higher in the low-dose than the high-dose group while *Verrucomicrobia* increased in the low-dose group but decreased in the high-dose group. *Bacteroidetes* decreased in the low-dose group but increased in the high-dose group.

Taken together, the phylum-level analysis suggests that perinatal TC-ART treatment has long-term influences in the gut microbiome composition, which is different between males and females.

To elucidate if the changes seen at the phyla level is also translated into the genus level, comparative analysis on the bacterial abundance at the genera level was conducted.

#### Genus level analysis

**FIGURE 5:**
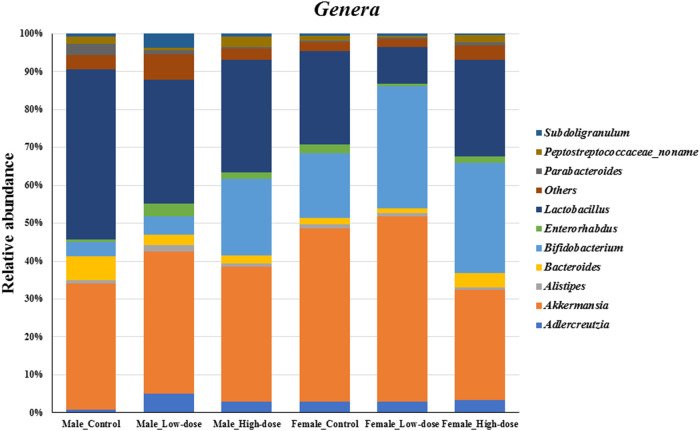
Changes in the fecal microbial composition at the *genera* level (top ten genus plus others) across different treatment groups (control, low-dose, and high-dose) in both male and female rats (n = 5 in each group).

At the genus level (Figure 5), the relative abundance data revealed notable differences between control and TC-ART treated groups in both males and females. In the control groups, *Lactobacillus*, *Akkermansia*, *Bifidobacterium*, and *Bacteroides* were among the most abundant genera. However, the relative abundances of these genera were altered upon treatment with TC-ART.

In males, the relative abundance of *Lactobacillus* decreased in both low-dose and high-dose treatment groups compared to the control. *Akkermansia* showed a slight increase in the low-dose group but decreased in the high-dose group. *Bifidobacterium* exhibited an increase in the treatment groups, with the high-dose group showing the highest relative abundance. *Bacteroides* and *Parabacteroides* decreased in both treatment groups.

In females, the relative abundance of *Lactobacillus* decreased in both low-dose and high-dose treatment groups, with the low-dose group showing a substantial decrease compared to controls. *Akkermansia* decreased in both treatment groups, with the high-dose group having the lowest relative abundance. *Bifidobacterium* increased in both treatment groups, with the low-dose group showing the highest relative abundance. *Bacteroides* and *Parabacteroides* increased in the high-dose group compared to the control.

The genera-level analysis reveals the differential impact of TC-ART on specific genus within the gut microbiome of males and females. The observed changes suggest that the drug modulates the relative abundances of key genera, such as *Lactobacillus*, *Akkermansia*, and *Bifidobacterium*, in a sex-specific manner. These alterations in genus-level composition contribute to the overall shifts observed at the phylum level.

To gain more granularity at the taxonomic level, the impact of TC-ART drug on different treatment groups was assessed by the heatmap analysis and Principal Coordinate Analysis (PCoA) at the species level.

#### Species level analysis

**FIGURE 6:**
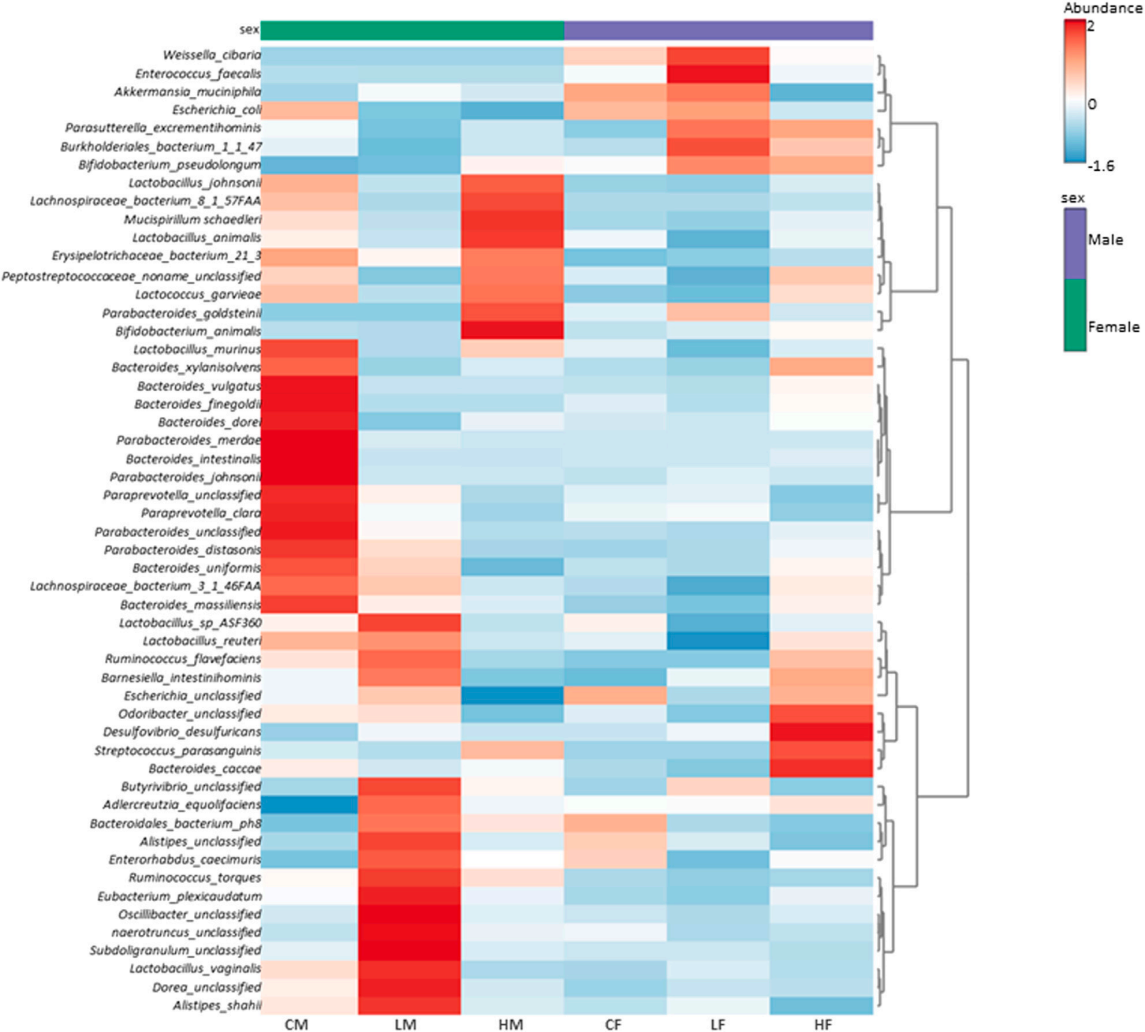
Heatmap illustrating the average (n = 5) relative abundance of bacterial species in the fecal microbiome for each treatment group (control, low-dose, and high-dose) in male and female rats. The color gradient from light to dark signifies low to high relative abundance. Vertical clustering represents the similarity in the abundance of different species among the treatment groups, with shorter branch lengths indicating greater similarity. Horizontal clustering shows the similarity of species abundance between treatment groups, with shorter branch lengths suggesting higher similarity between groups.

The heatmap in Figure 6 represents the average relative abundance of bacterial species in the fecal microbiome for each treatment group (control, low-dose, and high-dose; n = 5 per sex) in male and female rats (Supplementary Figure S1 displays the relative abundance for all animals in each group).

The control groups exhibit a distinct abundance profile compared to the treatment groups. The high-dose treated groups show an increase in the abundance of certain bacterial species, while the low-dose groups demonstrate an intermediate profile. These findings suggest that the treatment has a dose-dependent effect on the fecal microflora composition, with higher doses leading to more changes in the abundance of specific bacterial species.

The observed changes in microbial composition at the phylum, genus, and species levels are interconnected and reflect the taxonomic relationships among the affected bacteria. The decrease in *Firmicutes* at the phylum level might be primarily driven by the reduction in *Lactobacillus* species, which belong to this phylum. The increase in *Actinobacteria* can be largely attributed to the substantial increase in *Bifidobacterium pseudolongum*, a member of this phylum.

The sex-specific changes in *Akkermansia muciniphila*, the representative of the Verrucomicrobia phylum, directly contribute to the observed differences in *Verrucomicrobia* abundance between males and females. The increase in *Proteobacteria* in high-dose females can be linked to the slight increases in genera such as *Escherichia* and *Parasutterella*, which belong to this phylum.

The clustering patterns observed in the heatmap analysis further highlight the relationships among the affected species and their contribution to the overall changes in microbial composition. The co-clustering of various *Lactobacillus species* in males and their collective decrease with TC-ART underscore their shared response to the intervention. Similarly, the separate clustering of the control group in females emphasizes the impact of the drug on the female gut microbiome.

In conclusion, the perinatal exposure to TC-ART was associated with alterations in the gut microbial composition at multiple taxonomic levels, with sex-specific differences in adult rats. The changes observed at the phylum level are driven by the differential responses of specific genera and species, highlighting the intricate relationships within the gut microbiome.

#### Beta diversity analysis

##### Males

**FIGURE 7:**
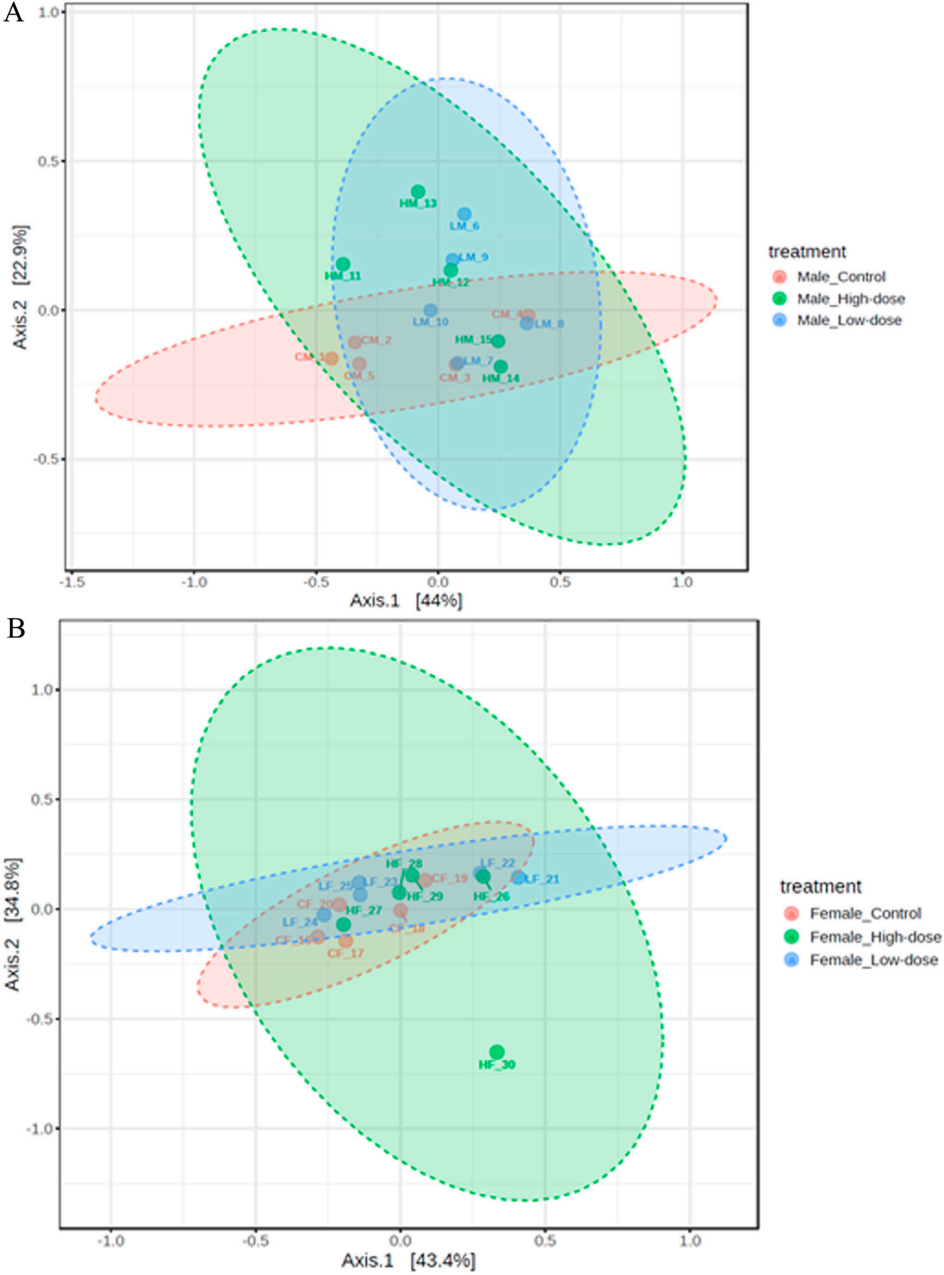
Principal Coordinate Analysis (PCoA) plots showing beta diversity in males **(A)** and females **(B)** rats across control (red points), low-dose (blue points), and high-dose groups (green points).

The PCoA plot for males (Figure 7A) reveals distinct clustering patterns related to the treatment groups. The control group (CM_1 to CM_5) forms two subclusters along Axis 1, indicating some within-group variation but overall separation from the treatment groups. This suggests that the untreated male samples have a distinct microbial community structure compared to those that received the treatments.

The low-dose group (LM_6 to LM_10) forms a relatively compact cluster, although it shows some variation along Axis 2. This cluster partially overlaps with both the control and high-dose groups, suggesting that the low-dose treatment induces a shift in the microbial community structure that is intermediate between the control and high-dose groups.

The high-dose group (HM_11 to HM_15) exhibits a separation from the control group, with samples spreading along both Axis 1 and Axis 2. This indicates that the high-dose treatment induces a shift in the microbial community structure compared to the untreated samples. However, the spread of the samples also suggests that there is considerable individual variation in the response to the high-dose treatment.

While there is a separation between the control group and the high-dose group, suggesting treatment-related changes, there is also some overlap, particularly between the low-dose group and the other groups. This overlap suggests that the treatment effect may not be as distinct for all individuals, and there could be other factors contributing to the variation within groups.

These results demonstrate that the treatments have an impact on the beta diversity of the male microbiome, with clustering patterns associated with each treatment group. The observed change across the treatment groups provides evidence for a dose-dependent response in the microbial community structure of males.

##### Females

The PCoA plot for females (Figure 7B) reveals a high degree of overlap among the control (CF_16 to CF_20), low-dose (LF_21 to LF_25), and high-dose (HF_26 to HF_30) groups. This overlap suggests that the treatments did not induce distinct shifts in the microbial community structure of females.

The control group samples tend to cluster towards the left side of the plot along Axis 1, but there is no clear separation between the control and treatment groups. This indicates that the untreated female samples do not have a markedly distinct microbial community structure compared to those that received the treatments.

The low-dose and high-dose groups are largely intermingled, with samples scattered throughout the plot. This lack of separation between the treatment groups suggests that increasing the treatment dose did not result in a consistent, dose-dependent shift in the microbiome composition of females.

The overall lack of clustering based on treatment groups in females contrasts with the patterns observed in males. While male samples showed distinct clustering and a gradient of change across treatment groups, female samples exhibit a high degree of overlap and no clear treatment-related patterns.

These results indicate that the treatments did not have a significant impact on the beta diversity of the female microbiome, as evidenced by the lack of distinct clustering patterns associated with the treatment groups. The overlap among the control and treatment groups suggests that factors other than the treatment itself may be driving the variation in the microbial community structure of females.

#### Effect of perinatal exposure to HIV TC-ART on SCFA levels in rat offspring

**FIGURE 8:**
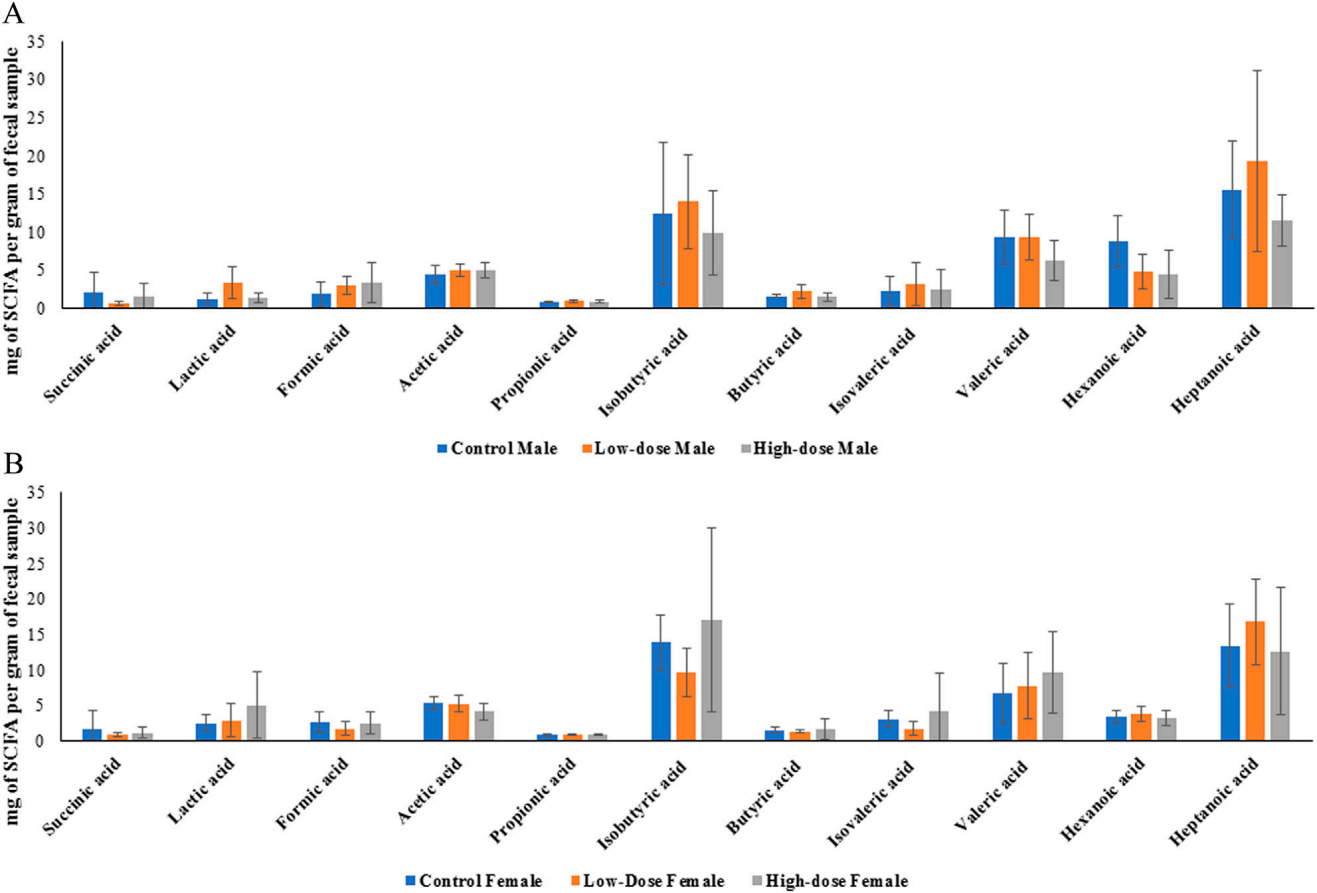
Bar graphs showing concentration of individual SCFA in control, low-dose, and high-dose groups for male **(A)** and female **(B)** rats. Each bar represents the mean ± SD of five observations (n = 5).

One prediction that the changes in microbiome abundance and composition as described above would be that their produced metabolites might also changed. Given that the metabolites derived from the microbiome can affect different biological processes, we then evaluated whether perinatal exposure to TC-ART would also impact the levels of SCFA produced by the bacteria. To this end, we compared the concentrations of various SCFAs between control, low-dose, and high-dose groups in both males and females. Figure 8 represents the average concentrations and standard deviations of each SCFA for the different treatment groups (control, low-dose, and high-dose) for male (A) and female (B) rats measured in fecal samples.

Data presented in Figure 8 showed no statistically significant differences between the groups. To better understand whether within the same sex the treatment had an effect, we next evaluated concentrations of each SCFA across different doses within the same sex. While we observed different trends in males and females, none of the data displayed statistically significant differences. Nevertheless, it is interesting that in both sexes the treatments tended to alter levels of lactic acid while the trends of other fatty acids were different in males and females (Figure 8).

#### Sex-dependent differences in SCFA concentrations

Among all the SCFAs analyzed, only hexanoic acid showed a statistically significant difference between sexes, with higher levels observed in control males compared to control females (p < 0.05). However, other SCFAs showed varying patterns between male and female rats across different treatment groups (represented by the considerable overlap of error bars in Figure 8).

Given the intricate relationship between gut microbiota, SCFAs, and mucosal immunity, we also examined Immunoglobulin A (IgA) levels in offspring. SCFAs are known to promote intestinal IgA responses, and investigating both parameters provide a more comprehensive view of how gestational ART exposure might influence the developing gut ecosystem.

#### Effect of perinatal exposure to HIV TC-ART on IgA levels in rat offspring

**FIGURE 9:**
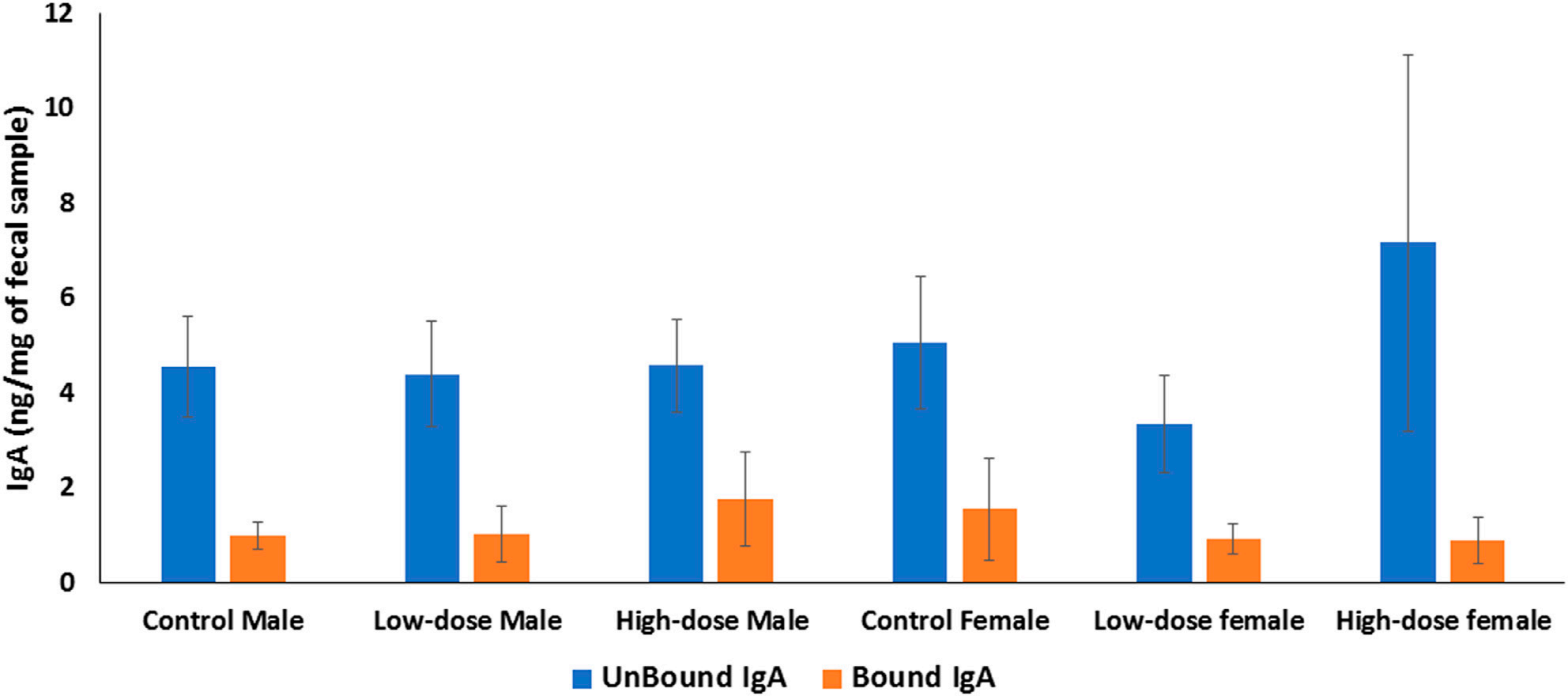
Bar graph showing quantification of IgA levels in the offspring feces. The level of IgA in rat feces is expressed as ng per mg of feces. IgA unbound (blue bars); bacteria-bound IgA (orange bars). Error bars represent standard deviation values (*n* = 5).

We quantified both secretory (fecal unbound) and bacterial-bound IgA and compared concentrations between control and treated groups in both males and females. The levels of unbound and bacteria-bound IgA were comparable between treated and control offspring, with no significant differences observed across the treatment groups (Figure 9). Similarly, no significant sex differences were observed between the levels of unbound or bacterial-bound IgA detected in the feces of adult offspring due to the perinatal exposure to HIV TC-ART.

## Discussion

It is estimated that 39 million people are infected with HIV and over the past few years [49], life expectancy of individuals living with HIV has improved significantly with the widespread usage of ART [50]. However, as the population of ART-treated individuals continues to grow, there is a pressing need to understand the long-term effects of ART exposure, particularly during important developmental stages such as during gestation [51–53], in the absence of the HIV.

Given the critical role of the gut microbiome in shaping immune function, metabolism, and neurodevelopment, investigation of the potential impact of ART on the gut microbiome and its consequences for the health of the offspring is warranted. The gut microbiome’s influence extends beyond the intestinal environment, playing roles in gut-brain communication, liver function, and cardiovascular health through complex bidirectional interactions known as the gut-brain axis, gut-liver axis, and gut-heart axis, respectively. In this study, we investigated the effects of perinatal ART exposure on the composition of the gut microbiome and their metabolites (SCFA’s) in aged rat offspring.

Our findings suggest that perinatal exposure to ART is associated with alterations in the gut microbiome composition in aged rat offspring at multiple taxonomic levels, with notable sex-specific differences. However, despite these changes in the gut microbiome composition, we did not observe statistically significant differences in SCFA levels across treatment groups or between sexes, which may be due to high individual variability.

Interestingly, our study did not find significant differences in the alpha diversity indices (Chao1 or Shannon) across treatment groups in either male or female rats. This suggests that the developmental ART exposure has not significantly affected the overall species richness or diversity of the fecal microbiome in the aged offspring at 12 months. The lack of significant differences in our study may be attributed to several factors, such as the subtle effects of gestational ART exposure on the fecal microbiome of the offspring, the long-term nature of the study allowing for microbial community recovery, and the high individual variability masking potential treatment effects [54]. However, it is important to consider that alpha diversity measures provide a broad overview of the microbial community structure and may not capture subtle changes in specific bacterial taxa [55, 56].

To gain a deeper understanding of the effects of developmental ART exposure on the fecal microbiome, we performed an integrated analysis of taxonomic levels, examining the changes at the phylum, genus, and species levels and their interconnectedness. Our results revealed dose-dependent and sex-specific alterations in the relative abundances of various bacterial taxa.

At the phylum level, we observed distinct differences between control and TC-ART treated groups in both males and females. In males, *Firmicutes* and *Bacteroidetes* exhibited a dose-dependent decrease, while *Actinobacteria* showed a dose-dependent increase. In females, the response was more complex, with *Firmicutes* decreasing in the low-dose group but increasing in the high-dose group, and *Actinobacteria* showing a dose-dependent increase. These findings suggest that TC-ART treatment may modulate the fecal microbiome composition in a dose- and sex-specific manner. Similar effects of antibiotics on the gut microbiome have been reported in previous studies [57–61].

The reduction in *Lactobacillus* species may increase offspring susceptibility to gastrointestinal disturbances and infections [62]. Conversely, the increase in *Bifidobacterium* species could offer some protective effects, given their association with improved immune function and metabolic health [63]. These alterations share similarities with findings from studies on early-life antibiotic exposure [64], suggesting that various early-life factors can induce long-lasting changes in gut microbiota composition. The sex-specific differences observed echo earlier findings [65] on sex-specific microbial patterns. This highlights the complex interplay between early-life exposures, sex hormones, and gut microbiome development. The species-level analysis, including the heatmap and clustering patterns, further confirmed the dose-dependent and sex-specific effects of TC-ART treatment on the gut microbiome composition. Specific bacterial species showed dose-dependent increases or decreases, while others exhibited sex-specific patterns of change. These species-level alterations drove the changes observed at the genus and phylum levels, highlighting the interconnectedness of taxonomic levels in the microbiome. Sex hormones such as estrogen and testosterone are known to influence gut microbiota composition and immune responses, potentially leading to distinct microbial community structures between males and females [66].

The observed changes in the gut microbiome composition may have important implications for extraintestinal organ functions, as discussed earlier regarding the gut microbiome’s role in immune function, metabolism, and gut-extraintestinal organ axes. *Lactobacillus* and *Bifidobacterium* species, which were affected by TC-ART treatment, are known for their probiotic properties and have been associated with various benefits, such as improved immune function, reduced inflammation, and protection against pathogens [67–70]. Conversely, a decrease in these beneficial bacteria has been linked to an increased risk of metabolic disorders, inflammatory bowel disease, and infections [71–73].

Moreover, the sex-specific alterations in key genera, such as *Akkermansia*, may have differential effects on health outcomes. *Akkermansia muciniphila*, which was more abundant in females, has been inversely associated with obesity, diabetes, and inflammation [74, 75]. The higher prevalence of this species in females may confer some protection against metabolic disorders, while its reduction in males may increase their susceptibility to these conditions [65, 76–78].

The dose-dependent effects of TC-ART treatment on specific bacterial species also warrant attention. For instance, the increase in *B. pseudolongum* in a dose-dependent manner may have positive implications for gastrointestinal tract, as this species has been shown to exert anti-inflammatory effects and improve gut barrier function [79–81]. However, the decrease in *Lactobacillus* species with increasing doses of TC-ART treatment may compromise the beneficial effects of these bacteria on the host.

The beta diversity analysis revealed sex-specific responses to developmental TC-ART exposure in the gut microbiome composition of rat offspring. Males exhibited distinct clustering patterns associated with treatment groups, indicating that gestational TC-ART exposure alters the gut microbiome composition in male offspring. The observed sex-specific alterations underscore the complex interplay between host factors, such as sex hormones, and the gut microbiome in response to early-life exposures [82]. In contrast to the male microbiome, the PCoA plot for females revealed a high degree of overlap among the control, low-dose, and high-dose groups, indicating that gestational ART exposure did not significantly alter the overall microbial community structure in female offspring. Colonization of bacterial community during early development may play a more dominant role in shaping the microbial community structure [65, 83, 84].

Several factors may contribute to the observed sex differences in response to gestational ART exposure, including hormonal influences [85, 86], gender-specific immune responses, genetic and epigenetic variations [87, 88]. In addition to the gut microbiome composition, we further investigated the impact of developmental ART exposure on the concentrations of SCFAs in the offspring. SCFAs are important microbial metabolites that play a role in maintaining gut homeostasis, regulating immune function, and influencing metabolic processes [89–91]. However, our analysis of SCFA and IgA concentrations revealed high variability within treatment groups, complicating the interpretation of the results. The high variability observed could be attributed to individual differences in gut microbiome composition and the complex nature of short‐chain fatty acid production and metabolism. In addition, the ongoing studies using metatranscriptomics analysis would help to understand if the observed microbiome changes result in different metabolic and immune responses between males and females. Moreover, integration of multi omics approaches such as metatranscriptomics, and metabolomics, would be valuable to better understand the impact of developmental exposure of TC-ART on the gut microbiome function and metabolic outputs.

## Conclusion

Our study showed that gestational and lactational exposure to TC-ART was associated with alterations in the fecal microbiome composition of aged rat offspring, with notable sex-specific differences. These changes were observed at various taxonomic levels and were characterized by dose-dependent and sex-specific patterns. Despite these changes in the fecal microbiome, we did not observe significant differences in IgA levels and SCFA concentrations across treatment groups or sexes. However, given the complex interplay between the microbiome and host factors, other functional consequences may exist that were not captured by these specific markers. Further investigations, including meta transcriptomics, could help to determine the impact of TC-ART on the gut microbiome and metabolic function in both males and females to account for sex differences.

## Data Availability

The datasets presented in this article will be available as per the guidelines of the U.S. Food and Drug Administration data sharing policy. Requests to access the datasets should be directed to Sangeeta.khare@fda.hhs.gov.

## Footnotes

1
https://clinicalinfo.hiv.gov/en/guidelines/perinatal/whats-new
