# Supplementary figures and images for: Effect of in utero and lactational exposure to antiretroviral therapy on the gut microbial composition and metabolic function in aged rat offspring
Source: Exp Biol Med (Maywood). 2025 May 21;250:10468. doi: 10.3389/ebm.2025.10468 (PMC12135209; doi:10.3389/ebm.2025.10468)

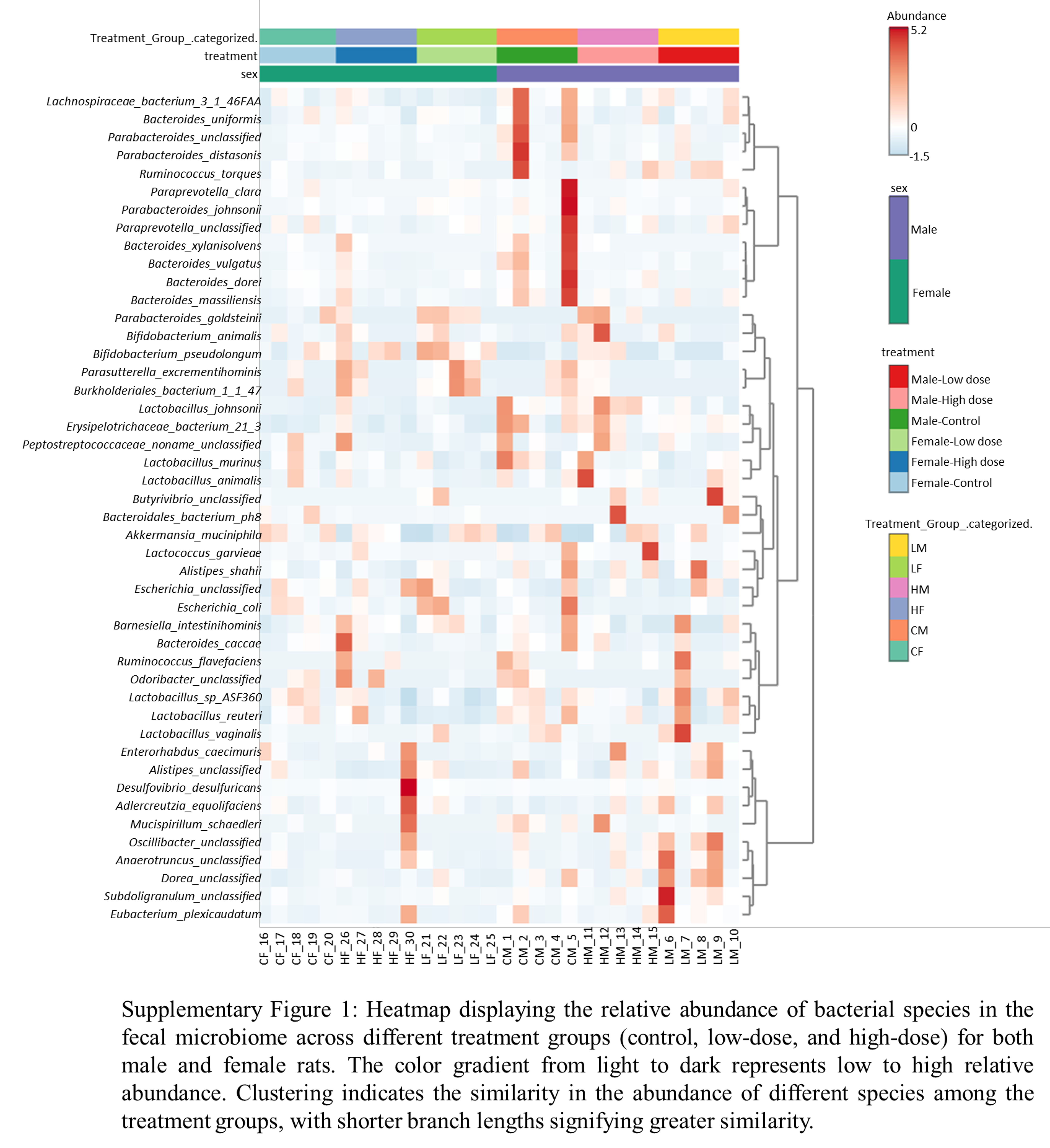

Supplement: Supplementary file 1 [file Image1.tif]
